# Double-Network Chitosan-Based Hydrogels with Improved Mechanical, Conductive, Antimicrobial, and Antibiofouling Properties

**DOI:** 10.3390/gels9040278

**Published:** 2023-03-29

**Authors:** Rahela Carpa, Anca Farkas, Cristina Dobrota, Anca Butiuc-Keul

**Affiliations:** 1 Department of Molecular Biology and Biotechnology, Faculty of Biology and Geology, Babeș-Bolyai University, 1 M. Kogălniceanu Street, 400084 Cluj-Napoca, Romania; rahela.carpa@ubbcluj.ro (R.C.); cristina.dobrota@ubbcluj.ro (C.D.); anca.keul@ubbcluj.ro (A.B.-K.); 2 Institute for Research-Development-Innovation in Applied Natural Sciences, Babeș-Bolyai University, 30 Fântânele Street, 400294 Cluj-Napoca, Romania; 3 Centre for Systems Biology, Biodiversity and Bioresource, Babeș-Bolyai University, 5–7 Clinicilor Street, 400006 Cluj-Napoca, Romania

**Keywords:** antibiofouling, antimicrobial, chitosan-based hydrogel, double-network hydrogel

## Abstract

In recent years, the antimicrobial activity of chitosan-based hydrogels has been at the forefront of research in wound healing and the prevention of medical device contamination. Anti-infective therapy is a serious challenge given the increasing prevalence of bacterial resistance to antibiotics as well as their ability to form biofilms. Unfortunately, hydrogel resistance and biocompatibility do not always meet the demands of biomedical applications. As a result, the development of double-network hydrogels could be a solution to these issues. This review discusses the most recent techniques for creating double-network chitosan-based hydrogels with improved structural and functional properties. The applications of these hydrogels are also discussed in terms of tissue recovery after injuries, wound infection prevention, and biofouling of medical devices and surfaces for pharmaceutical and medical applications.

## 1. Introduction

Hydrogels have received special attention due to their promising properties, such as softness, endowment, and high capacity to hold water [1,2]. The hydrophilic functional groups in the polymer’s backbone allow it to retain water, whereas crosslinks between network catena prevent it from dissolving [3,4]. Hydrogels can be created through either a physical or a chemical crosslinking process. Based on these two fundamental advances, there are numerous preparation paths for obtaining hydrogel structures. Physical crosslinkers primarily consist of host–guest complexes, hydrophobic–hydrophobic, electrostatic, ionic, precipitation, and stereo complex coactions, followed by the development of polymer networks [5,6,7,8]. Physically crosslinked hydrogels have significant advantages in diverse biological applications because they lack chemical crosslinkers, which could potentially cause unforeseeable and harmful side effects to the tissues. They are also more biocompatible. However, their reversible construction, low mechanics, and stability severely limited their range of applications [9,10,11,12]. The mechanical stability of physical crosslinking-produced hydrogels decreases as conditions change, such as temperature or pH [13,14,15].

In contrast to physical crosslinkers, chemical crosslinkers are created by covalently joining polymer chains. The network obtained is the result of highly efficient synthetic techniques such as free radical polymerization, click chemistry, Schiff’s base reaction, and photopolymerization [16,17]. Because of the irreversible connections between polymeric chains, chemically crosslinking hydrogels have stable constructions and superior mechanics, making them suitable for the tissue engineering sector. Chitosan-based hydrogels are promising biomaterials with a wide range of applications such as tissue engineering, carriers for the controlled delivery of drugs, and even genes, with the goal of increasing the drug concentration at a specific application site and coating layers to prevent biofilm formation on medical devices and surfaces in clinical and industrial environments. Chitosan is one of the valuable natural polymers used for biological applications due to its chemical properties and inherent antimicrobial activity. Moreover, chitosan is easily obtained by deacetylation of chitin from different sources such as crustaceans, fungi, and insects, which ensures an acceptable price for the obtained products [18]. The subsequent physical and chemical functionalizing of chitosan or development of multi-network structures confer improved mechanical and biological properties to the novel polymer structure and multiple applications. They have a three-dimensional and porous framework creating a biocompatible extracellular matrix for the attachment and proliferation of the cells. Crosslinking at chitosan polymers is required to improve chitosan’s properties for drug delivery, such as stability and endurance. Different types of chitosan-based hydrogel networks exist depending on how the chitosan is prepared and crosslinked [19,20].

When chitosan is combined with other biomaterials that are either synthesized via covalent and non-covalent linkages or obtained from natural sources, a variety of multifunctional hydrogels are formed.

Chitosan is a natural biopolymer, a non-toxic biodegradable compound derived from chitin via deacetylation under alkaline conditions [21,22,23]. This carbohydrate has a straight-chain chemical structure that includes -(1,4)-linked 2-amino-2-deoxy-D-glucopyranose and 2-acetamino-2-deoxy-D-glucopyranose. Chitosan’s properties are conferred by three included functional groups: an amino group and two hydroxyl groups (primary and secondary), which are inserted at positions C-2, C-3, and C-6, respectively [24,25]. Thus, chitosan is more chemically reactive in comparison with chitin.

Chitosan can be synthesized homogeneously [26] or heterogeneously [27,28,29] obtained through a series of N-deacetylation reactions beginning with chitin. Its properties and molecular weight differ depending on the source. Chitosan is a more soluble deacetylation product than chitin [30]. Chitin and chitosan are antibacterial, antioxidant, antifungal, and prebiotic compounds with minor side effects characterized by their versatility, abundance, plasticity, biodegradability, biocompatibility, and non-toxicity [31,32,33,34,35,36,37]. All of this gives them a distinct advantage as biotechnology compounds.

Furthermore, chitosan depolymerization generates bioactive substances with antibacterial properties. It is renewable, non-toxic and biodegradable and has excellent antimicrobial properties, an excellent film-forming capability, and excellent chelation and absorption properties. As a result, chitosan has a wide range of applications such as biomedical engineering, bioremediation, hydraulic engineering, food industry [38], biotechnology, cosmetics, textile and paper industries, as well as agriculture [24,39,40,41,42]. Chitosan is a naturally occurring polymer with excellent biocompatibility and biodegradability that is already widely used in biomedical applications [43,44].

Chitosan is a promising material for biomedical applications due to a variety of properties. Chitosan is currently used in drug and gene delivery, enzyme confinement, surface modification, wound healing, dialysis membranes, and bone regeneration [45,46,47,48,49,50]. Much research has been conducted in order to emphasize that chitosan has antibacterial, antifungal, antitumor, immunoadjuvant, anticholesteremic, and antithrombogenic features and the ability to increase the re-epithelization and acceleration of wound healing [51,52,53,54,55].

Double-network (DN) hydrogels are the most promising biomaterials for modern medicine, having both a high water content and high mechanical properties. The structural characteristic of DN hydrogels is the special network structure consisting of two components: the minor component, which is represented by crosslinked polyelectrolytes that form the rigid skeleton, and the major component, which comprises poorly crosslinked neutral polymers with ductile characteristics [56]. Due to the large range of chemical compounds and methods used to develop such biomaterials, in this article, we highlight the current state of the evolving methods for preparing double or multi-network chitosan-based hydrogels in order to enhance their mechanical and conductive properties and harvest their antibacterial and antibiofouling properties for various pharmaceutical and medical applications.

## 2. Chemical Changes for Chitosan Functionalization

**Figure 1:**
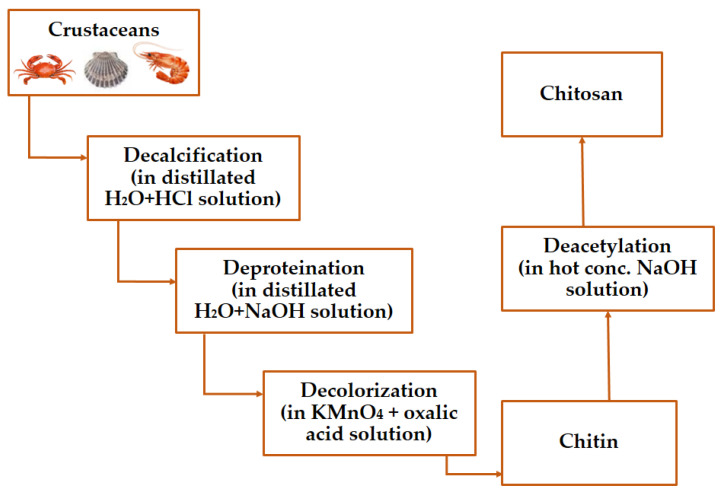
Chitosan extraction methods from crustaceans.

Chitin is a natural polysaccharide from the shells of various taxonomic groups such as turtles, crustaceans, and insects [57,58,59], or is the result of a fungal fermentation process [60,61,62]. Chitosan is a derivative of chitin, and it is gathered with minerals such as calcium carbonate, proteins, and residual pigments. To obtain pure chitin, the raw source is demineralized with acid and then proteins are removed with alkali (Figure 1). Demineralization and deproteination can also be accomplished using enzymatic, chemical, and fermentation methods [18,62]. Chitin and chitosan can both be produced using traditional methods (Figure 1).

Alternative processing techniques reduce processing times and the amount of alkali required to deacetylate chitin. Examples include sequential alkali treatments, saturated steam flash treatment, thermomechanical processes in a cascade reactor run at low alkalinity, dielectric heating, and intermittent water washing. Enzymatic deacetylation has been demonstrated in specific fungi and bacteria [63]. Another study also describes a microwave technique for converting chitin nanowhiskers into chitosan nanoscaffolds [34].

Chitosan is composed of (1–4)-linked D-glucosamine with irregularly positioned N-acetylglucosamine groups, depending on the degree of polymer deacetylation. Alkaline hydrolysis is commonly used to perform repeated deacetylation [64].

The components of chitin and chitosan contain an amino group that facilitates the chemical modification of these polymers. As a result, chitin and chitosan have proven to be useful and excellent materials for different applications as biomaterials in the pharmaceutical, medical, chemical, cosmetical, agricultural, and sustainability sectors. Numerous studies have been conducted regarding the properties, variations, and applications of these polymers [25,65].

Chitosan contains three essential functional groups: an amino group (-NH_2_ at C-2), a primary hydroxyl group (-OH at C-6), and a secondary hydroxyl group (OH at C-3) [66,67]. These active groups allow the chemical modification of the chitosan chain [68]. Such processes are carried out by substituting or reacting with active hydroxyl or amino groups within the chitosan molecule [69]. These groups form intermolecular hydrogen bonds without interfering with polymerization, allowing for the modification of chitosan chains that are copolymerized by crosslinking with other polymers. This results in a variety of composite scaffolds that are appealing candidates for bone repair and reconstruction.

Chitosan has some important properties, including a low-cost source, easy processing, rapid and complete biodegradability, antibacterial activity, nonantigenicity, high conductivity, high porosity with the proper pore size distribution, controlled drug delivery, and biocompatibility with most human tissues, that make chitosan very attractive for many applications [70,71,72,73]. The physicochemical properties of chitosan can be improved through chemical derivatization.

**Figure 2:**
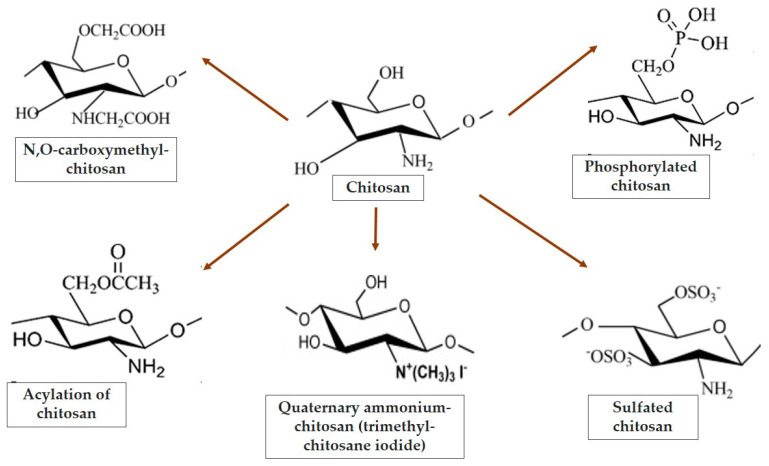
Examples of chitosan functionalization.

Chitosan-based hydrogels have been made functional by encapsulating bioactive compounds and delivery to the target sites at appropriate doses and for the desired durations. Bioactive agents, proteins, amino and nucleic acids, and drugs can all be encapsulated in chitosan-based reactive hydrogels to create intelligent delivery systems and bone regeneration. There are numerous widely used methods for modifying the chemical and thus physical properties of chitosan (such as carboxymethylation, phosphorylation, sulphation, quaternization, esterification, grafting, or crosslinking) (Figure 1).

a. Crosslinking. Chitosan can be used in a variety of ways, including composites, hydrogels, membranes, and chitosan nanoparticles. The disadvantages of some of these biomaterials include cytotoxicity, degradability, and low mechanical properties. These issues can be overcome by crosslinking or stabilizing such materials. Crosslinking is a widely used method for modifying this substance’s physical and chemical properties [74]. Several studies have shown that chitosan molecules can be linked with a wide range of other compounds [74,75,76]. Carbodiimide-mediated crosslinking of chitosan nanoparticles and collagen increases collagenase resistance and thus reduces its biodegradability [77]. Crosslinking with citric acid produced dopamine-modified chitosan hydrogels for use in neural tissue engineering. Dopamine’s high density of crosslinking points allowed the hydrogel to have a rigid structure and significant mechanical strength through crosslinking [78]. The properties conferred by chitosan crosslinking result in frames with reduced degradability, immunogenicity, and toxicity, in addition to biocompatibility. As a result, chitosan is suitable for regenerative medicine and a valuable bioresource for tissue engineering [79].
b. Carboxymethylation is a widely used chemical synthesis method. The chitosan derivative under the most scrutiny is carboxymethylchitosan, an amphoteric polymer whose solubility is determined by the pH. Chitosan carboxymethylation reactions can occur on the amino and hydroxyl groups, yielding N-, O-, or N,O-carboxymethylchitosan (Figure 2) [80,81,82]. Through a reductive alkylation at the amino group at C-2, glyoxylic acid can be used to obtain N-carboxymethylchitosan, while monochloroacetic acid can be used to obtain O-carboxymethylchitosan. As a result, amphoteric ether derivatives are formed [80,82,83]. N,O-carboxymethylchitosan is formed by the simultaneous addition of carboxymethyl groups to the amino and hydroxyl loci of the glucosamine part. This reaction was carried out in isopropanol at 50 °C by mixing chitosan with sodium hydroxide and monochloroacetic acid, and the scheme of this process was improved in such a way that the solubility of this compound in water was significantly increased [82,84].

Chitosan carboxymethylated by-products are water-soluble and have antibacterial, antimutagenic, and antioxidant properties [82,85,86,87]. Because of their rheological properties, O-carboxymethylchitosan and N,O-carboxymethylchitosan are valuable viscosity inducers, as well as amphoteric electrolytes with antitumoralproperties. These derivatives have hemostatic activity and are suitable for filtration membrane construction [82,88,89]. Carboxymethylated chitosan can be used to create hydrogels with appropriate adhesion and pH-dependent swelling behavior [87,89]. All of the above attributes make chitosan derivatives suitable for applications such as pharmaceutics, wound care, cosmetics, tissue engineering, biomedicine, metal absorption appliances, and food storage [80,82,90].

c. Quaternary ammonium chitosan derivatives. Another major class of chitosan derivatives is quaternary ammonium salts. In alkaline solutions containing methyl iodide, the amino groups of chitosan can be quaternized to varying degrees. N-methylpyrrolidinone represents the first step of the reaction [91]. Following that, the reaction proceeds with chloroacetyl chloride in dimethysulfoxide as reagents, followed by pyridine or amino-pyridine in a third reaction step [92].

Chitosan quaternary derivatives are soluble in neutral and weakly alkaline solutions, and they have antioxidant and antifungal properties as well as low toxicity [80,92,93]. Furthermore, because these polymers have better absorption and mucoadhesion than chitosan, they are suitable for gene- and drug-delivery applications [80,91,94].

The excellent properties of quaternized chitosan-based multiple complexes have facilitated the testing of a variety of current applications, including virus adsorption [95], wound cure [96], and antimicrobial treatment [97,98].

Quaternized chitosans have excellent mechanical properties that regulate bioadhesion, mucoadhesion, and biodegradation, making them effective antibacterial compounds. When tested on human fibroblasts, they were found to be safe for use as joining tissues, and biocompatibility studies in rats revealed no negative effects when implanted subcutaneously. As a result, it has been established that the dual quaternary chitosan/chitosan fiber is a suitable bioactive material for tissue reconstruction, wound healing, and drug delivery schemes [99].

Attempts to create networks from chitosan quaternary salts were only successful when two components were used: polymers such as poly(vinyl alcohol) [100], poly(lactic acid) [101], or polyvinylpyrrolidone [97] plus adjuvants such as graphene [94]. This study shows that quaternized chitosan-based nanofibers are promising biomaterials and has prompted additional research to improve their design.

d. Phosphorylated chitosan. There are numerous methods for producing phosphorylated chitosan derivatives. Chitosan can be phosphorylated with phosphorous pentoxide or orthophosphoric acid via thermal treatment, depending on the applications. Furthermore, phosphorylated chitosan can be efficiently produced via the reaction of phosphorus pentoxide and methanesulfonic acid [58,102,103]. Increased hydroxyl phosphorylation on chitosan at carbon 3 and carbon 6 improves the bacterial cell wall. Chitosan can be mono- or disubstituted, depending on the chemical reaction (Figure 2) [91].

Water-soluble phosphorylated chitosan has several wound-healing properties, including hemostatic properties, metal chelating bonding, antioxidant, anti-inflammatory, and osteoinductive properties, as well as angiogenic and bactericidal influence [104,105,106]. Because it avoids drug release in the acidic region of the stomach, this chitosan derivative can also be used for the oral administration of drugs [91,105]. In diabetic rats, phosphorylated chitosan accelerates wound healing [106].

e. Alkylated chitosans are important polysaccharide amphiphilic polymers that can be produced through a variety of chemical reactions. The most common is the chitosan reaction with acyl chlorides and anhydrides [91]. Chitosan can be acylated in pyridine/chloroform or methanesulfonic acid with decanoyl chloride or hexanoyl chloride to produce N,O-acyl chitosans [81,91]. Another method of acylation of chitosan involves the use of p-nitrobenzoic acid, myristic acid, or hydrochloric acid in an acetone–water complex [107]. The N-acylation of chitosan with acetic anhydride can regenerate chitin [108]. Chitosan acylation results in chelation, aggregation, and the formation of polymers with biological functionality [89,107]. O-acyl chitosan was developed as a biodegradable coating material, and N,O-acyl chitosan shows antifungal activity in relation to the length of the acyl chain [91,109].
f. Sulfated chitosan. Several methods exist for producing sulfated chitosan, including the use of sulfuric acid or chlorosulfonic acid. It can be carried out in various conditions and reaction media such as tetrahydrofuran, dimethylformamide, or formic acid, or it can be microwave irradiated [91]. Depending on the sulfation reaction conditions, S-chitin is mono-, di-, or tri-substituted and is frequently N,O-disubstituted (Figure 2) [110,111].

Sulfated chitosans, or chitosans with a film-forming capacity, are valuable derivatives due to their biological activities. They have antithrombotic and anticoagulant properties similar to heparin, as well as antiviral, antibacterial, antioxidant, and enzyme-inhibitory properties. They are antioxidants with anti-obesity properties due to adipogenesis inhibition [112,113,114,115,116,117]. Water-insoluble antitumoral drugs can be solubilized in sulfated chitosan micelles, indicating that this polymer is suitable as a drug carrier in specific systems [112,118]. In addition, sulfated chitosan has high metal absorption properties, making it useful for metal ion recovery systems [119].

**Figure 3:**
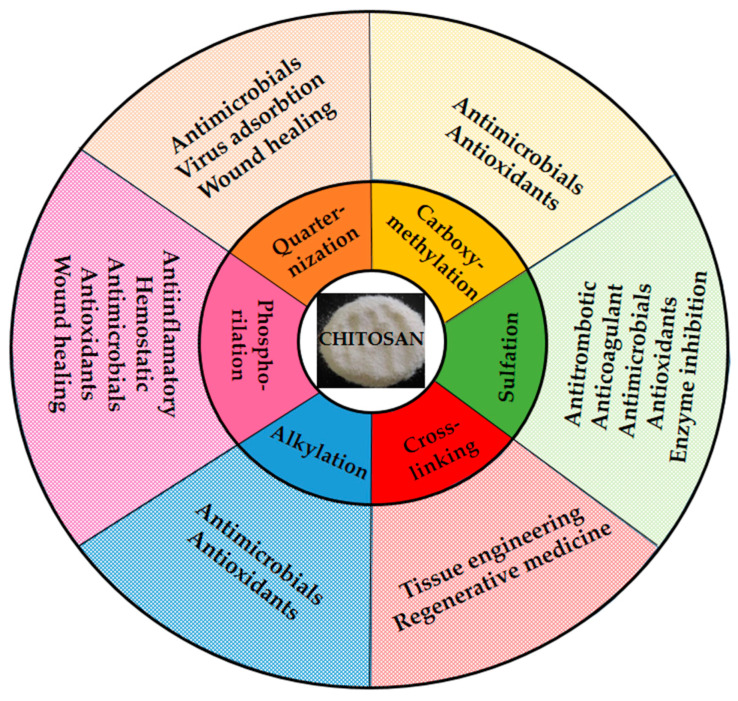
Applications of chitosan derivatives.

Moreover, numerous additional derivatives have been produced and involved in several practical sectors, such as thiolated, acetylated, or sulfonamide ones (Figure 3).

## 3. Synergistic Action of Chitosan in Combination with Other Active Agents

Chitosan-based hydrogels are formulated in a variety of shapes and contain a wide range of biomaterials [120], which are widely used for different biomedical applications [121] due to their non-toxicity, biodegradability, biocompatibility, stimulus responsiveness, and antibacterial activity [122]. Yet, one of the most significant drawbacks impacting chitosan utilization is its low solubility [123] and poor mechanical qualities due to the high water content [124]. Because of its cationic nature, chitosan can interact electrostatically with sodium sulfate to generate gel particles [125] and with hydrophobic chemicals to form amphiphilic particles with self-assembly and encapsulating capabilities [126]. Covalent and non-covalent chitosan modification was used to improve chitosan-based hydrogels while avoiding environmental concerns [127]. The use of plasticizers often increases flexibility, but it is followed by a decrease in biopolymer film strength [128], making concurrently improving strength and flexibility a crucial task. The development of double-network (DN) hydrogels could provide a solution to these issues. Consequently, DN hydrogels were created by incorporating a crosslinking network into a polymer network, which resulted in better mechanical characteristics, stretchability, and shape recovery [129]. The first network is usually hard and crosslinked skeletons, and the second network is poorly crosslinked ductile substances [130]. Consequently, DN hydrogels have two interpenetrating networks, the first of which is hard and quickly ruptures, and the second of which is soft and ductile, assuring hydrogel stretchability [131]. Because of its inherent antibacterial qualities, chitosan is one of the most valuable chemicals employed as the initial network, but other polysaccharides could also be used [132,133].

DN hydrogels are constructed usually by chemically–chemically crosslinking, although hybrid physically–chemically crosslinked DN hydrogels display improved self-recovery, resistance, and biocompatibility [134,135,136,137].

Improved solubility of chitosan-based hydrogels was achieved by the grafting of the quaternary ammonium group on the chitosan chain (QCS), which also improved the antibacterial properties [138,139]. A glycidyl methacrylate complex QCS (QCSG) was further developed for wound dressing due to the high versatility of QCS to polymerize with various materials, creating injectable hydrogels in the wound [140]. In other experiments different hydrogels containing glycidyl methacrylate functionalized QCSG, gelatin methacrylate (GM), and graphene oxide (GO) were prepared for the healing of wounds infected with MRSA, having improved mechanical, electrical, and photothermal properties [141].

Short chitosan chains were attached to polyacrylamide (PAM) hydrogels to improve their mechanical characteristics, and these hydrogels were subsequently treated with a base solution to produce transparent DN hydrogels. The opaque DN hydrogels were generated by treating these composite hydrogels with saline solution [136]. Furthermore, the polymerization of the PAM network and polyaniline ensured the formation of DN hydrogels that respond quickly to low pressure [142]. By combining two types of chitosan polymers (catechol-modified methacryloyl chitosan and methacryloyl chitosan) and simultaneously crosslinking carbon–carbon double bonds and catechol-Fe3+ chelation, a novel double-crosslinking DN injectable hydrogel with improved adhesion properties and antibacterial activity was obtained [143].

Crosslinking chitosan-based hydrogels with b-glycerolphosphate disodium salt pentahydrate yielded another functionalization. In vivo, the novel DN hydrogel demonstrated acceptable outcomes for healing wounds infected with resistant bacteria, but in vitro findings were not sufficient [144].

Another hydrogel dressing for wound healing with a suitable swelling ratio, biocompatibility, self-healing, and mechanical qualities was created via non-covalent bonding of cordycepin and chitosan [145].

By combining quaternized chitosan-g-polyaniline with poly(ethylene glycol)-co-poly(glycerol sebacate) (PEGS-FA), the antimicrobial activity and cytocompatibility of chitosan-based hydrogels were improved [146]. Because of its superior biocompatibility and antibacterial activity against *E. coli* and *Staphylococcus aureus*, this copolymer was employed as an injectable dressing [147].

Crosslinking of konjac glucomannan and CS, as well as the insertion of silver nanoparticles (AgNPs), resulted in a nanocomposite hydrogel dressing with suitable rheological characteristics and biocompatibility [148]. Chitosan hydrogels loaded with AgNPs were effective against both Gram + and Gram − bacteria and usually showed reduced toxicity toward mammalian cells [149]. However, several toxic effects were reported depending on the method of administration. Oral administration of AgNPs in rats showed Ag distribution in an order of blood > liver > kidneys [150]. Intravenous administration showed that AgNPs were first accumulated in the liver and spleen, and then in other organs [151].

The combination of chitosan with GO improves the physicochemical and optical properties of chitosan-based hydrogels [152]. Due to the synergistic effect of the components, a hydrogel produced by crosslinking aminated-GO, chitosan, and cellulose had a high antimicrobial activity against *S. aureus* despite the components’ poor antibacterial capabilities [153]. A non-cytotoxic polymer based on chitosan formulated with methylcellulose was developed and used as a nanofiller and drug carrier [154,155]. Polyvinyl alcohol (PVA), polyethylene oxide (PEO), polyglycolic acid (PGA), polycaprolactone (PCL), and polyvinylpyrrolidone (PVP) were combined with CS to create GO-CS nanocomposite fibrous membranes for wound treatments [156,157]. The GO-coated CS/PLA (poly lactic acid) nanofibrous scaffolds also showed an increased surface roughness, hydrophilicity, and antibacterial activity against *E. coli* and *S. aureus*, facilitating cell proliferation and wound healing [158].

A bioinspired dual bionic adhesive chitosan-based hydrogel grafted with methacrylate (CS-MA), dopamine (DA), and N-hydroxymethyl acrylamide (NMA) with sealant capabilities, hemostatic activity under wet conditions, antibacterial qualities, and biocompatibility was recently produced. This polymer is a promising biomaterial for hemostasis and wound healing because it replicates the polysaccharide adhesin of a staphylococcal biofilm and the 3,4-dihydroxy-L-phenylalanine (Dopa) of mussel adhesive protein [159,160].

Another technique for creating multifunctional hydrogels was to modify chitosan with dodecyl, which acts as an anchor in the cell membrane and provides hemostasis and tissue adhesion. Furthermore, because of the intrinsic antibacterial activity of chitosan, the innovative hydrogel was employed for wound healing [161], which is assisted by sprayable hydrogels and bilayer dressings based on nanofiber and hydrogel. Some of these biomaterials for wound healing are also based on gelatin grafting with methacrylate [162].

Chitosan-based DN and triple-network (TN) antimicrobial hydrogels with zwitterionic sulfopropylbetaine (PDMAPS) as the second network and nonionic poly (2-hydroxyethyl acrylate) (PHEA) as the final network were produced. Because of their biocompatibility, nonfouling, and mechanical qualities, these hydrogels can be employed for wound treatment and other biomedical applications [163]. Another DN crosslinked polysaccharide-based hydrogel for skin wound healing was created, which consists of collagen peptide-functionalized carboxymethyl chitosan and oxidized methacrylate sodium alginate (SA) [164]. Chitosan or thiolated chitosan functionalization with poly (ethylene glycol) diacrylate (PEGDA) ensured the development of DN hydrogels with good mechanical and adhesion qualities to promote skin regeneration [165].

In comparison to covalent crosslinked hydrogels, DN self-healing hydrogels were created on the chitosan matrix by ionic crosslinking and hydrogel bonding. The reaction of chitosan with poly(acrylic acid) results in the formation of DN hydrogel via Fe^f^ ion coordination and hydrogen bonds that ensure regeneration when the hydrogel is disrupted, with the crosslinking points being re-formed based on the hydrogel’s dual network. This hydrogel also shows superior mechanical and electrical properties [166].

## 4. Biomedical Applications of DN Chitosan Hydrogels

The chemical structure of chitosan determines its functional qualities and thus its primary applications. The ratio of the two groups, N-acetyl D-glucosamine and glucosamine, determines the degree of deacetylation of the biopolymer. The physicochemical and biological features such as crystallinity, solubility, hydrophilicity, degradation, reactivity, adsorptive capacity, and cell responsiveness are determined by the molecular weight and degree of deacetylation [167,168,169,170]. N-acetyl glucosamine can create hydrogen bonds and hydrophobic interactions, which help to stabilize the molecule by providing stiffness and enhancing its structural features. The amino groups of glucosamine protonate in acidic circumstances, and the polymer becomes cationic, allowing interactions with a wide range of molecules. Its positive charge is responsible for its antibacterial and biological activity via contact with negatively charged cell membranes [65,171]. Furthermore, additional chemical changes targeting the reactive amino and hydroxyl groups result in a diverse set of derivatives with enhanced functionality [92].

### 4.1. Biomedical Applications of DN Chitosan Hydrogels with Antimicrobial and Antibiofouling Properties

Biofouling of biomaterials is a critical challenge, since protein fouling, microbial colonization, and biofilm development may impair medical devices and implants, leading to a failure of intervention or treatment and even to life-threatening complications. Their preliminary treatment with various antimicrobial agents and antibiotic-eluting coatings was extensively investigated, although there is a risk of colonization with resistant bacterial strains. Antibiotic-loaded biomaterials designed for the prolonged release of drugs raise serious concerns for their weak efficacy and even more, for their possible contribution to enhancing biofilm formation and selecting resistant mutants [172]. Non-antibiotic alternatives, such as cationic polymers [173], antimicrobial non-adhesive coatings [174,175], biomaterial-assisted delivery of bacteriophages [176], antimicrobial peptides [177], and antimicrobial enzymes [178], have improved the ability to prevent biofouling and even to treat antibiotic-resistant and recurring infections. Antibacterial biomaterials and delivery systems of non-antibiotic therapeutics allow targeted delivery at the infection site, reducing the potential systemic adverse effects [179].

**Figure 4:**
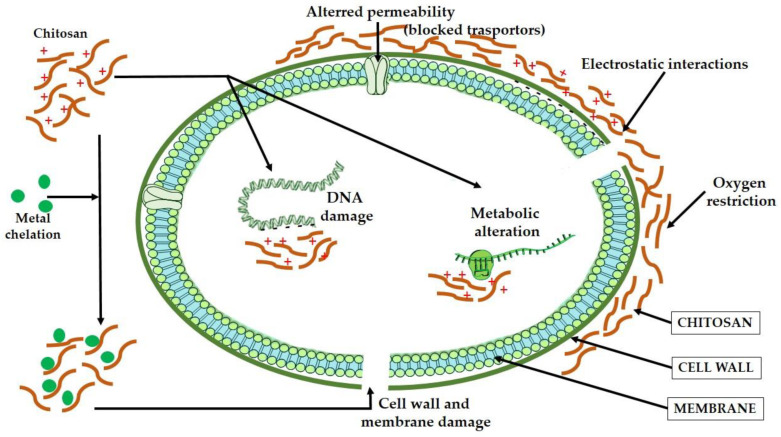
Antimicrobial mechanisms of chitosan-based materials.

Chitosan, as a polycationic polymer, exhibits inherent antibacterial action through a variety of mechanisms, as shown in Figure 4: (i) cell membrane disruption caused by the electrostatic interaction of its positively charged amino groups with the negatively charged microbial cell membrane; (ii) interference with microbial metabolism caused by low-molecular-weight chitosan; (iii) inhibition of microbial growth caused by metal chelation; and (iv) nutrient and oxygen restriction caused by a polymeric film absorbed on the cell surface [180].

It is generally accepted that antibacterial activity of the chitosan and its derivatives is largely influenced by the molecular weight of chitosan, the degree of deacetylation of chitosan, the chitosan source, the concentration of chitosan, the pH value, the temperature, the cell growth phase, the type and concentration of composite materials, and the type of microorganisms [181]. However, the inherent antimicrobial activity of chitosan is low; thus, its complexation with different compounds is compulsory to improve its biological properties [36].

The combined features of multi-network chitosan hydrogels confer several antibiofilm and antibacterial actions. Antimicrobial activity against bacteria and fungi may be increased in multi-network chitosan hydrogels. Polyvinyl alcohols, polyacrylamides, zwitterionic materials, and other synthetic polymers used as a second network improve hydrogel self-recovery, biocompatibility, biodegradability, antibacterial, and antifouling properties.

DN poly(N-(2-hydroxyethyl)acrylamide)/chitosan hydrogels crosslinked with citrate or sulphate prepared by Zhang et al. [182] were described as “repelling and killing bacteria”. The biomaterials displayed anti-protein-adsorption properties, with up to 5% fouling after soaking in PBS for 0.5 h. L929 cell adhesion was negligible after 7 days and cytotoxicity was also insignificant. Bacterial adhesion and biofilm formation were inhibited, especially on DN chitosan hydrogel crosslinked with citrate. Similar antibacterial effects were observed against *Escherichia coli* and *Staphylococcus aureus* [182]. DN and TN chitosan hydrogels with zwitterionic polymers and polyacrylates also showed limited cell adhesion, cytotoxicity, and biodegradability. A significant decrease of up to 95.5% in cell adhesion of mouse embryo fibroblasts NIH 3T3 was observed compared to polystyrene controls. The cytotoxicity was low and the TN hydrogel had lower cytotoxicity on NIH 3T3 and macrophage cells than the DN hydrogel. No weight loss at all lysozyme concentrations was observed for any of the multi-network gels after 160 h. The antimicrobial activity was evaluated using the inhibition of growth after 24 h. It was 99.4% for *E. coli* ATCC25922 and 99.96% for *S. aureus* ATCC6538 on DN and 89.7% for *E. coli* ATCC25922 and 91.6% for *S. aureus* ATCC6538 on TN hydrogels [163]. Double noncovalent network chitosan/hyperbranched polyethylenimine/Fe^3+^ films exhibited distinct antimicrobial activity against *E. coli* and against *S. aureus*. The inhibition zone diameters varied between 12.38 and 13.07 mm [183]. In conclusion, DN hydrogels are able to inhibit protein fouling and biofilm formation of both Gram + as well as Gram − bacteria.

### 4.2. Biomedical Applications of DN Chitosan Hydrogels with Improved Mechanical and Conductive Properties

Promising candidates for various applications including tissue engineering, implantable medical devices, wearable electronics, and controlled drug delivery, conductive hydrogels are able to transform external stimuli into a variation of electrical signals [184]. Living organisms conduct electricity mostly using ions, while inorganic matter conducts electricity mostly using electrons. The two systems, natural and synthetic, function through distinct ionic and electronic circuits that are coupled at human–machine interfaces [185]. The engineering of conductive hydrogels is based on electron conductors and/or ions into hydrogel matrices [186]. Conductive hydrogels are usually fabricated by adding conductive materials (graphite, carbon nanotubes), conducting polymers (polyaniline), free ions, or liquid metals to the biopolymer network. For instance, mussel-inspired composite chitosan-based hydrogels were prepared for electroactive tissue engineering. Thus, Jing et al. [187] used chitosan/graphene oxide while Liang et al. [188] employed gelatin-grafted dopamine, chitosan, and polydopamine-coated carbon nanotubes. Guo et al. [189] investigated self-healable injectable hydrogels based on dextran-graft-aniline tetramer-graft-4-formylbenzoic acid and N-carboxyethyl chitosan. These conductive biomaterials proved their potential as cell-delivery vehicles and scaffolds for skeletal muscle repair. PEGylated chitosan grafted with aniline was developed as a cell-delivery carrier for cardiac cell therapy [190]. Chitosan-based thermosensitive hydrogels with Au nanoparticles were prepared for applications in cardiac tissue engineering [191]. Due to their biocompatibility, electron-conductive hydrogels are widely employed in bioengineering applications. Ion conductive hydrogels may need additional strategies (such as hydrophobic coating) to limit water permeability, ion diffusibility and leakage. Moreover, hydrophylic hydrogels cannot easily incorporate conductive hydrophobic polymers. However, the main drawbacks of early conductive hydrogels are their limited elasticity, stretchability, strength, and toughness [192]. Therefore, DN hydrogels were engineered by introducing a dynamic crosslinking network into a polymer to achieve conductivity. Because of the intrinsic softness, deformability, biocompatibility, and electrical properties, they are ideal candidates as flexible biosensors and actuators.

One significant disadvantage of chitosan-based hydrogels, especially those used in pharmaceutical and medical applications, is their poor strength. The multi-network design structure is one technique for increasing the hydrogel mechanical characteristics. As mentioned previously, the term “double-network” hydrogel refers to two interpenetrating networks with opposing mechanical properties formed by one natural and one synthetic polymer. The optimal mechanical strength is obtained at a specific ratio of the two networks, which is controlled by the cross-connecting density [193]. The hard and brittle network serves as a sacrificial bond to effectively release energy, and the soft and ductile network preserves the hydrogel integrity during the deformation process [131,194,195]. The difficult preparation method and fussy performance regulation of DN hydrogels typically limit their use in many industries [196].

Recent research has shown that the physical and mechanical properties of DN hydrogels can be widely customized by manipulating the hydrogel compositions and regulated by varying the chitosan content. DN chitosan hydrogels with better mechanical characteristics were successfully produced using a variety of aqueous solutions, polymerization initiators, and conditions (Table 1). Jiang et al. [195], for example, created a chitosan-based DN hydrogel by dissolving chitosan in an AlCl_3_·6H_2_O solution and adding dissolved acrylic amide, acrylic acid, N,N′-methylenebisacrylamide, and 2-hydroxy-4′-(2-hydoxyethoxy)-2-methylpropiophenone. UV polymerization was used to create the hybrid crosslinked hydrogel. The dynamic ionic interaction between Al^3+^ and the macromolecular chains results in properties such as high toughness, stretchability, and excellent form recovery. Using comparable physically–chemically crosslinking procedures, a number of novel cytocompatible chitosan-based DN and TN hydrogels were also created. Zwitterionic sulfopropylbetaine was chosen by Zou et al. [163] as the second network, whereas nonionic poly (2-hydroxyethyl acrylate) was employed as the final network. Besides having outstanding mechanical properties, multinetwork gels also have good antibacterial, cytocompatible, and antifouling capabilities, which are important for biological applications such as wound dressing.

Zhang et al. [17] used the “one pot” approach to create an ultra-high-strength poly(N-(2-hydroxyethyl)acrylamide/chitosan hydrogel in a -ketoglutaric acid solution, which was then exposed to UV light. Citrate or sulfate ions were used to crosslink the chains of chitosan. The biomaterial was suggested for use in biomedical procedures such as the construction of artificial connective tissues, implantable biosensors, and bandages for wound healing.

Gan et al. [196] used a two-step freezing/thawing and immersion procedure to create a physically crosslinked poly(vinyl alcohol)-(2-hydroxypropyltrimethyl ammonium chloride chitosan) DN hydrogel without the use of organic solvents or harmful crosslinking chemicals. Because of the reversible ionic networks, the hydrogels developed had excellent elasticity, high strength, strong self-recovery, and anti-fatigue performance. The authors demonstrated that the structures and mechanics of DN hydrogels could be altered flexibly by varying the immersion period or the concentration of the trisodium nitriloacetate solution, providing direction for the design and synthesis of environmentally friendly DN hydrogels. To create a DN hydrogel with numerous hydrogen bonding contacts, the freezing–heating alternative treatment was applied to a chitosan-poly(vinyl alcohol) solution, followed by incubation in alkaline conditions. Superior compressive, tensile, recoverability, and anti-swelling qualities, in combination with cell compatibility, showed that the hydrogel could promote cell attachment and wound healing, making it suitable for tissue engineering repair [197].

The integration of mechanical performances with high conductivity to meet the needs for flexible sensors and other practical applications was also examined. DN electron-conductive hydrogels with a high fracture energy were designed by generating polypyrrole nanorods in hydrogel matrices consisting of polyacrylamide and chitosan [198]. A strain sensor capable of detecting the movement of human joints through electrical signals was built using a conductive composite hydrogel made of polyaniline, double-bonded modified chitosan, and acrylamide. This strain sensor is anticipated to be used in wearable health monitoring and multi-functional robot skin [199]. Further conductive DN hydrogels were produced by Zeng et al. [200] using in situ polymerization of acrylamide in a carboxymethyl chitosan aqueous solution, followed by immersion in a ferric chloride solution. The biomaterials showed acceptable mechanical characteristics, such as adequate tensile strength, prominent stretchability, and excellent fatigue resistance. These characteristics, along with their high conductivity, allowed the biomaterials to accurately and repeatedly track the motions of body joints, such as the finger and wrist, demonstrating their suitability for use with flexible sensors.

The hydrogels created by Cong et al. [184] had a DN made of dynamically crosslinked chitosan and a flexible polyacrylamide network with polyaniline doping. These hydrogels had high tensile stress, elastic modulus, tensile strength, and tensile strain. Their impressive antifreezing abilities, ionic and electric conductivity, sensitive sensing, and excellent UV resistance, in addition to their good flexibility, point to their potential for use in harsh environments. Recently, a noncovalent crosslinking technique was used to create novel chitosan/hyperbranched polyethyleneimine and chitosan/hyperbranched polyethyleneimine/Fe^3+^ films [183]. After a small amount of iron ions was added, the film’s tensile strength increased while the strain reduced. The notion of using such films as biosensors for iron detection came from the usage of switches to raise and decrease the fluorescence of DN films using hyperbranched polyethyleneimine and Fe^3+^. Another ionic/electronic dual conductive hydrogel was produced by Zhang et al. [142] by merging the chemically crosslinked polyacrylamide and the physically crosslinked carboxymethyl chitosan-grafted-polyaniline/Ag^+^ network. For wearable strain sensors and self-powered strain sensors with potential applications in human health, the DN hydrogel demonstrated great stretchability, reproducible adhesiveness, high sensitivity, and consistent electrical performance.

**Table 1:** DN chitosan-based hydrogels with improved mechanical and physical properties and their proposed applications.

DN Chitosan-Based Hydrogels	Aqueous Solution, Polymerization Conditions	Hydrogel Properties	Proposed Application	Ref.
DN: Chitosan/p(acrylic amide-acrylic acid)-Al^3+^	AlCl_3_·6H_2_O UV 365 nm, 8 W	Tensile strength 0.54 MPa; elongation at break 2203.7%	Load-bearing artificial soft tissues	[195]
DN: Chitosan/zwitterionic sulfopropylbetaine TN: poly(2-hydroxyethyl acrylate)	Acetic acid α-ketoglutaric acid initiator; N,N′-methylenebisacrylamide crosslinker; UV 365 nm, 8 h	DN: Compressive stress 84.7 MPa; tensile stress 292 kPa, TN: Compressive stress 81.9 MPa; tensile stress 384 kPa	Wound dressing	[163]
DN: Chitosan/poly(N-(2-hydroxyethyl)acrylamide	“One-pot” method with α-Ketoglutaric acid; UV 365 nm, 8 h; Soaked into saturated sodium citrate or sodium sulfate solution 20 min	Tensile strength 3.8 MPa; elastic modulus 0.6 MPa; self-recovery; fatigue resistance	Artificial connective tissues, implantable biosensors, and wound dressings	[182]
DN: 2-Hydroxypropyltrimethyl ammonium chloride chitosan/poly(vinyl alcohol)	Trisodium nitriloacetate Freezing/thawing (−20 °C for 12 h/25 °C for 12 h) and immersion (30–330 min)	Tensile stress 4.14 MPa; compression stress 73.55 MPa; elongation at break 832%	Tissue scaffolds, environment areas, and actuators	[196]
DN: Chitosan/poly (vinyl alcohol)	Freezing (−20 °C)/heating (25 °C) alternate treatment (3 cycles); Incubation in alkaline KOH/urea solution at 45 °C	Compressive stress 60%–230 KPa; tensile stress 152 KPa–360%; recoverability 90.77% after five cycles	Tissue engineering	[197]
DN: Chitosan/polyacrylamide/polypyrrole nanorods	Acqueous solution UV 365 nm, 2.8 mW/cm^2^, 5 min FeCl_3_ solution at 4 °C 12 h	Compressive strength 6.5 MPa;Tensile strength 0.8 MPa; elongation at break 260%; conductivity 0.3 S/m.	Wearable electronic devices, wound dressings, sensors, and electrostimulated drug-release systems	[198]
DN: Double bond modifed chitosan/polyaniline and acrylamide	HCl solution Immersion in FeCl_3_ solution, thermal oxidative polymerization	Tensile strength 0.3 MPa; electrical conductivity 6.97 S/m; strain sensitivity—gauge factor 15.9	Wearable health monitoring and multi-functional robot skin	[199]
DN: Carboxymethyl chitosan/polyacrylamide	Aqueous solution Immersion in FeCl_3_ solution	Tensile strength 440 kPa; stretchability 715%; toughness 1658 kJ m⁻^3^; conductivity 3.1 S/m.	Flexible sensors	[200]
DN: Chitosan/polyacrylamide doped with polyaniline	2, 2′-azobis(2-methyl-propionamidine) dihydrochloride—initiator; 50 °C 12 hImmersion in (NH_4_)_2_SO_4_ and HCl	Tensile stress 2.62 MPa; elastic modulus 253.79 kPa; tensile strength 2.62 MPa; tensile strain up to 927%; ionic and electric conductivity; sensitive sensing; freezing resistance; UV resistance	Devices for extreme environments	[184]
DN: chitosan/hyperbranched polyethylenimine/Fe^3+^	Acetic acid solution Iron ions Thermal crosslinking at 60 °C and drying at 45 °C	Tensile stress 42 MPa; tensile strain up to 72%; UV resistance; strong fluorescence emission	Biosensors	[183]

## 5. Conclusions and Perspectives

Hydrogels containing chitosan and chitosan derivatives that are crosslinked with physical or chemical processes are promising biopolymers with remarkable properties. Because of their superior biocompatibility and biodegradability, as well as low immunogenicity and toxicity, chitosan-based formulations for biomedical and other applications have been developed. DN chitosan hydrogels are emerging biomaterials with improved self-recovery, resistance, flexibility, biocompatibility, antimicrobial, and antifouling properties due to the synergistic effect of the components. Chitosan-based hydrogels are formulated in a variety of shapes using various aqueous solutions, polymerization initiators, and conditions. The different qualities of multifunctional hydrogels can be customized using a variety of materials as the second network.

Multi-network chitosan-based hydrogels can be modulated with a wide range of mechanical and conductive properties and explored as flexible biosensors for human health monitoring, smart actuators, artificial tissues, wearable displays, drug-delivery systems, and implants for cell regeneration. Depending on the application, further promising strategies employ improvement of their performances and the development of engineered platforms such as three-dimensional biomimetic scaffolds and dynamic scaffold-based microenvironments. In the context of the antibiotic resistance crisis, engineered biomaterials such as multi-network chitosan-based hydrogels are demonstrating the potential for a progressive alternative in the antimicrobial approach.

## Data Availability

Not applicable.
